# Dinitro­sylbis[tris­(4-chloro­phen­yl)phosphane]iron

**DOI:** 10.1107/S1600536811001693

**Published:** 2011-01-22

**Authors:** Myron W. Jones, Douglas R. Powell, George B. Richter-Addo

**Affiliations:** a Department of Chemistry and Biochemistry, University of Oklahoma, 101 Stephenson Parkway, Norman, OK 73019-5251, USA

## Abstract

The title dinitrosyl iron diphosphane complex, [Fe(NO)_2_(C_18_H_12_Cl_3_P)_2_] or Fe(NO)_2_ *L*_2_ [*L* = P(C_6_H_4_-*p*-Cl)_3_] belongs to the family of metal dinitrosyl compounds with the general formula Fe(NO)_2_(*L*)_*x*_, referred to collectively as dinitrosyl iron compounds (DNICs). The iron atom is tetra­hedrally coordinated by two phosphane ligands and two NO groups with Fe—N—O bond angles of 178.76 (15) and 177.67 (14)°.

## Related literature

For the preparation of the starting compound, Fe(NO)_2_(CO)_2_, see: Eisch & King (1965 ▶). For the structures of some related dinitrosyl complexes, see: Li *et al.* (2003 ▶); Atkinson *et al.* (1996 ▶); Li Kam Wah *et al.* (1989 ▶); Albano *et al.* (1974 ▶). For general information on metal nitrosyl chemistry, see: Richter-Addo & Legzdins (1992 ▶).
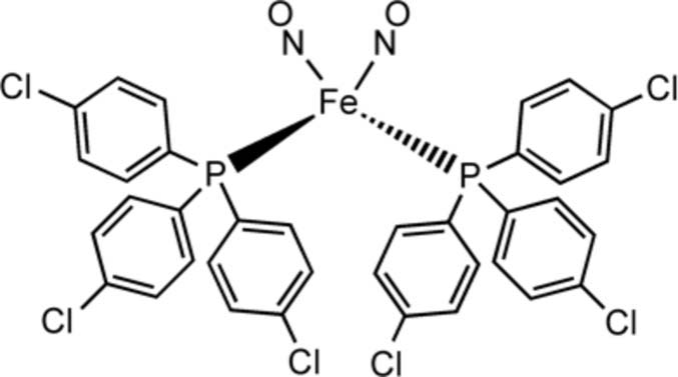


## Experimental

### 

#### Crystal data

[Fe(NO)_2_(C_18_H_12_Cl_3_P)_2_]
*M* *_r_* = 847.06
Monoclinic, 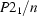

*a* = 10.340 (3) Å
*b* = 35.025 (10) Å
*c* = 10.589 (3) Å
β = 108.399 (8)°
*V* = 3638.9 (18) Å^3^
*Z* = 4
Mo *K*α radiation
μ = 0.98 mm^−1^
*T* = 100 K
0.38 × 0.19 × 0.04 mm

#### Data collection

Bruker APEX CCD diffractometer
Absorption correction: multi-scan (*SADABS*; Sheldrick, 2001 ▶) *T*_min_ = 0.702, *T*_max_ = 0.966
24943 measured reflections
7073 independent reflections
6527 reflections with *I* > 2σ(*I*)
*R*_int_ = 0.023

#### Refinement

*R*[*F* ^2^ > 2σ(*F* ^2^)] = 0.028
*wR*(*F* ^2^) = 0.072
*S* = 1.00
7073 reflections
442 parameters
H-atom parameters constrained
Δρ_max_ = 0.39 e Å^−3^
Δρ_min_ = −0.21 e Å^−3^

### 

Data collection: *SMART* (Bruker, 2007 ▶); cell refinement: *SAINT* (Bruker, 2007 ▶); data reduction: *SAINT*; program(s) used to solve structure: *SHELXTL* (Sheldrick, 2008 ▶); program(s) used to refine structure: *SHELXTL*; molecular graphics: *SHELXTL*; software used to prepare material for publication: *SHELXTL*.

## Supplementary Material

Crystal structure: contains datablocks I, global. DOI: 10.1107/S1600536811001693/hg2779sup1.cif

Structure factors: contains datablocks I. DOI: 10.1107/S1600536811001693/hg2779Isup2.hkl

Additional supplementary materials:  crystallographic information; 3D view; checkCIF report

## Figures and Tables

**Fig. 1.**
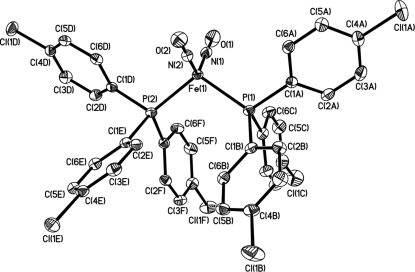
The molecular structure of the title compound. Hydrogen atoms have been omitted for clarity. The displacement ellipsoids are drawn at the 50% probability level.

## supplementary crystallographic information

### Comment

The molecular structure of the title compound is shown in Fig. 1. The molecule possesses a distorted tetrahedral geometry around the iron center. The iron is bound to two nitrosyl groups *via* the nitrogen atoms and to two phosphane ligands *via* the phosphorous atoms. The Fe(NO)_2_ group exhibits an *attracto* conformation where the bond angles O···Fe···O < N—Fe—N (Richter-Addo & Legzdins, 1992). The N—Fe—N bond angle is 127.78 (7)° and the interphosphane angle, P—Fe—P, is 106.80 (3)°. The Fe—N—O bond angles are 178.76 (15)° and 177.67 (14)°. For the structures of some related complexes, see: Li *et al.*, 2003, Atkinson *et al.*, 1996, Li Kam Wah *et al.*, 1989, and Albano *et al.* 1974.

### Experimental

A colorless toluene solution (4 ml) of P(C_6_H_4_-*p*-Cl)_3_ (126 mg, 0.35 mmol) was charged with Fe(NO)_2_(CO)_2_ (20 µ*L*, 0.18 mmol) (Eisch & King, 1965). The light red solution was heated and stirred under nitrogen. After 20 min the color of the solution had changed to black/brown. The reaction was allowed to proceed for 3.5 h until the infrared spectrum indicated the absence of characteristic carbonyl stretching frequencies for Fe(NO)_2_(CO)_2_ (ν_CO_ = 2090 cm^-1^ and 2040 cm^-1^). The reaction mixture was filtered through celite under N_2_ and the solvent was subsequently removed under vacuum. Isolated yield of the Fe(NO)_2_*L*_2_ compound: 33%. IR (toluene, cm^-1^): ν_NO_ = 1722 s and 1682 s. ^31^P{^1^H} NMR (CDCl_3_): δ 60.9 (*s*) referenced to 85% H_3_PO_4_. Suitable crystals for X-ray diffraction studies were grown by slow evaporation of a chloroform solution of the complex under nitrogen at ambient temperature.

### Refinement

H atoms were placed using known geometry with C—H (phenyl = 0.95 Å, methyl = 0.98 Å). Displacement parameters of phenyl H atoms were set to 1.2 times the isotropic equivalent for the bonded C.

### Crystal data

**Table:**

[Fe(NO)_2_(C_18_H_12_Cl_3_P)_2_]	*F*(000) = 1712
*M**_r_* = 847.06	*D*_x_ = 1.546 Mg m^−^^3^
Monoclinic, *P*2_1_/*n*	Mo *K*α radiation, λ = 0.71073 Å
Hall symbol: -P 2yn	Cell parameters from 8898 reflections
*a* = 10.340 (3) Å	θ = 3.0–26.8°
*b* = 35.025 (10) Å	µ = 0.98 mm^−^^1^
*c* = 10.589 (3) Å	*T* = 100 K
β = 108.399 (8)°	Plate, red
*V* = 3638.9 (18) Å^3^	0.38 × 0.19 × 0.04 mm
*Z* = 4	

### Data collection

**Table:**

Bruker APEX CCD diffractometer	7073 independent reflections
Radiation source: fine-focus sealed tube	6527 reflections with *I* > 2σ(*I*)
graphite	*R*_int_ = 0.023
ω scans	θ_max_ = 26.0°, θ_min_ = 3.0°
Absorption correction: multi-scan (*SADABS*; Sheldrick, 2001)	*h* = −11→12
*T*_min_ = 0.702, *T*_max_ = 0.966	*k* = −43→43
24943 measured reflections	*l* = −13→13

### Refinement

**Table:**

Refinement on *F*^2^	Primary atom site location: structure-invariant direct methods
Least-squares matrix: full	Secondary atom site location: difference Fourier map
*R*[*F*^2^ > 2σ(*F*^2^)] = 0.028	Hydrogen site location: inferred from neighbouring sites
*wR*(*F*^2^) = 0.072	H-atom parameters constrained
*S* = 1.00	*w* = 1/[σ^2^(*F*_o_^2^) + (0.038*P*)^2^ + 2.2*P*] where *P* = (*F*_o_^2^ + 2*F*_c_^2^)/3
7073 reflections	(Δ/σ)_max_ = 0.001
442 parameters	Δρ_max_ = 0.39 e Å^−^^3^
0 restraints	Δρ_min_ = −0.21 e Å^−^^3^

### Fractional atomic coordinates and isotropic or equivalent isotropic displacement parameters (Å^2^)

**Table:**

	*x*	*y*	*z*	*U*_iso_*/*U*_eq_	
Fe1	0.39351 (2)	0.360112 (6)	0.72317 (2)	0.01475 (7)	
N1	0.22480 (15)	0.35677 (4)	0.67163 (15)	0.0211 (3)	
O1	0.10393 (13)	0.35496 (4)	0.63323 (16)	0.0374 (4)	
N2	0.49768 (15)	0.36032 (4)	0.87866 (14)	0.0183 (3)	
O2	0.56832 (14)	0.36016 (4)	0.99132 (12)	0.0286 (3)	
P1	0.42791 (4)	0.414501 (12)	0.62695 (4)	0.01567 (10)	
C1A	0.37536 (16)	0.45624 (5)	0.70326 (17)	0.0168 (3)	
C2A	0.33928 (18)	0.49081 (5)	0.63462 (18)	0.0225 (4)	
H2A	0.3363	0.4925	0.5442	0.027*	
C3A	0.30787 (18)	0.52255 (5)	0.69749 (19)	0.0244 (4)	
H3A	0.2825	0.5459	0.6505	0.029*	
C4A	0.31395 (17)	0.51983 (5)	0.82958 (18)	0.0215 (4)	
C5A	0.34768 (18)	0.48586 (5)	0.89891 (18)	0.0218 (4)	
H5A	0.3503	0.4843	0.9892	0.026*	
C6A	0.37756 (17)	0.45423 (5)	0.83540 (17)	0.0197 (3)	
H6A	0.3999	0.4308	0.8824	0.024*	
Cl1A	0.27894 (5)	0.559906 (13)	0.91015 (5)	0.03403 (12)	
C1B	0.60313 (16)	0.42712 (5)	0.63916 (16)	0.0170 (3)	
C2B	0.66819 (18)	0.45927 (5)	0.70917 (17)	0.0215 (4)	
H2B	0.6199	0.4757	0.7498	0.026*	
C3B	0.80257 (18)	0.46732 (5)	0.71992 (18)	0.0242 (4)	
H3B	0.8465	0.4891	0.7683	0.029*	
C4B	0.87206 (17)	0.44347 (5)	0.65970 (18)	0.0217 (4)	
C5B	0.81105 (17)	0.41094 (5)	0.59145 (17)	0.0209 (4)	
H5B	0.8602	0.3945	0.5518	0.025*	
C6B	0.67730 (17)	0.40302 (5)	0.58234 (17)	0.0193 (3)	
H6B	0.6350	0.3807	0.5365	0.023*	
Cl1B	1.03788 (5)	0.454627 (16)	0.66918 (6)	0.03857 (13)	
C1C	0.33064 (17)	0.42091 (5)	0.45080 (16)	0.0185 (3)	
C2C	0.38994 (19)	0.42696 (5)	0.35119 (18)	0.0230 (4)	
H2C	0.4864	0.4279	0.3735	0.028*	
C3C	0.3091 (2)	0.43157 (5)	0.21917 (18)	0.0265 (4)	
H3C	0.3502	0.4351	0.1514	0.032*	
C4C	0.1692 (2)	0.43096 (5)	0.18746 (18)	0.0269 (4)	
C5C	0.10724 (19)	0.42500 (6)	0.28448 (18)	0.0270 (4)	
H5C	0.0107	0.4247	0.2618	0.032*	
C6C	0.18857 (18)	0.41947 (5)	0.41529 (18)	0.0237 (4)	
H6C	0.1469	0.4146	0.4818	0.028*	
Cl1C	0.06876 (6)	0.438658 (17)	0.02359 (5)	0.04204 (14)	
P2	0.46109 (4)	0.312563 (12)	0.61848 (4)	0.01492 (9)	
C1D	0.40364 (16)	0.26589 (5)	0.65914 (16)	0.0165 (3)	
C2D	0.33939 (17)	0.23830 (5)	0.56611 (17)	0.0210 (4)	
H2D	0.3153	0.2440	0.4738	0.025*	
C3D	0.31013 (18)	0.20251 (5)	0.60686 (18)	0.0228 (4)	
H3D	0.2654	0.1838	0.5431	0.027*	
C4D	0.34701 (17)	0.19451 (5)	0.74143 (18)	0.0202 (4)	
C5D	0.41099 (18)	0.22145 (5)	0.83637 (17)	0.0206 (4)	
H5D	0.4361	0.2155	0.9285	0.025*	
C6D	0.43760 (17)	0.25712 (5)	0.79447 (17)	0.0198 (3)	
H6D	0.4797	0.2760	0.8587	0.024*	
Cl1D	0.31377 (5)	0.149324 (12)	0.79346 (5)	0.02760 (11)	
C1E	0.64188 (16)	0.30117 (5)	0.65467 (16)	0.0171 (3)	
C2E	0.74298 (17)	0.32280 (5)	0.74395 (16)	0.0191 (3)	
H2E	0.7184	0.3444	0.7857	0.023*	
C3E	0.88021 (18)	0.31309 (5)	0.77269 (17)	0.0218 (4)	
H3E	0.9491	0.3278	0.8341	0.026*	
C4E	0.91421 (17)	0.28181 (5)	0.71054 (17)	0.0213 (4)	
C5E	0.81523 (18)	0.25973 (5)	0.62067 (17)	0.0216 (4)	
H5E	0.8405	0.2384	0.5782	0.026*	
C6E	0.68019 (18)	0.26927 (5)	0.59438 (17)	0.0204 (4)	
H6E	0.6118	0.2540	0.5345	0.025*	
Cl1E	1.08460 (4)	0.269062 (13)	0.74297 (5)	0.02791 (11)	
C1F	0.39668 (17)	0.31651 (5)	0.43779 (16)	0.0170 (3)	
C2F	0.48224 (17)	0.32239 (5)	0.36092 (16)	0.0182 (3)	
H2F	0.5780	0.3201	0.4010	0.022*	
C3F	0.42994 (18)	0.33155 (5)	0.22701 (17)	0.0209 (4)	
H3F	0.4891	0.3360	0.1757	0.025*	
C4F	0.29012 (18)	0.33409 (5)	0.16937 (17)	0.0214 (4)	
C5F	0.20195 (18)	0.32781 (5)	0.24218 (17)	0.0230 (4)	
H5F	0.1061	0.3292	0.2007	0.028*	
C6F	0.25575 (17)	0.31942 (5)	0.37631 (17)	0.0209 (4)	
H6F	0.1961	0.3156	0.4275	0.025*	
Cl1F	0.22499 (5)	0.344935 (16)	0.00079 (4)	0.03281 (12)	

### Atomic displacement parameters (Å^2^)

**Table:**

	*U*^11^	*U*^22^	*U*^33^	*U*^12^	*U*^13^	*U*^23^
Fe1	0.01424 (12)	0.01603 (12)	0.01523 (13)	−0.00054 (9)	0.00643 (9)	0.00098 (9)
N1	0.0199 (8)	0.0189 (7)	0.0271 (8)	0.0002 (6)	0.0109 (6)	0.0042 (6)
O1	0.0146 (7)	0.0378 (8)	0.0593 (10)	0.0004 (6)	0.0111 (6)	0.0135 (7)
N2	0.0207 (7)	0.0162 (7)	0.0194 (8)	0.0014 (5)	0.0085 (6)	0.0007 (5)
O2	0.0294 (7)	0.0345 (7)	0.0183 (7)	0.0021 (6)	0.0026 (6)	0.0010 (5)
P1	0.0134 (2)	0.0180 (2)	0.0163 (2)	−0.00109 (16)	0.00563 (16)	0.00173 (16)
C1A	0.0116 (8)	0.0172 (8)	0.0221 (8)	−0.0010 (6)	0.0060 (6)	0.0008 (6)
C2A	0.0216 (9)	0.0238 (9)	0.0234 (9)	−0.0014 (7)	0.0089 (7)	0.0058 (7)
C3A	0.0213 (9)	0.0186 (8)	0.0322 (10)	0.0007 (7)	0.0067 (7)	0.0068 (7)
C4A	0.0157 (8)	0.0182 (8)	0.0288 (9)	0.0000 (6)	0.0043 (7)	−0.0033 (7)
C5A	0.0213 (9)	0.0218 (9)	0.0209 (9)	−0.0003 (7)	0.0048 (7)	0.0003 (7)
C6A	0.0176 (8)	0.0194 (8)	0.0211 (9)	0.0013 (7)	0.0049 (7)	0.0036 (7)
Cl1A	0.0377 (3)	0.0223 (2)	0.0359 (3)	0.00912 (19)	0.0028 (2)	−0.00645 (19)
C1B	0.0149 (8)	0.0199 (8)	0.0163 (8)	−0.0008 (6)	0.0051 (6)	0.0038 (6)
C2B	0.0198 (9)	0.0237 (9)	0.0222 (9)	0.0001 (7)	0.0083 (7)	−0.0018 (7)
C3B	0.0199 (9)	0.0250 (9)	0.0260 (9)	−0.0058 (7)	0.0050 (7)	−0.0047 (7)
C4B	0.0126 (8)	0.0293 (9)	0.0234 (9)	−0.0024 (7)	0.0060 (7)	0.0018 (7)
C5B	0.0167 (8)	0.0264 (9)	0.0211 (9)	0.0023 (7)	0.0079 (7)	0.0020 (7)
C6B	0.0190 (8)	0.0182 (8)	0.0199 (8)	−0.0020 (7)	0.0052 (7)	0.0014 (6)
Cl1B	0.0177 (2)	0.0528 (3)	0.0489 (3)	−0.0128 (2)	0.0158 (2)	−0.0172 (2)
C1C	0.0192 (8)	0.0175 (8)	0.0180 (8)	−0.0016 (7)	0.0049 (7)	0.0016 (6)
C2C	0.0208 (9)	0.0272 (9)	0.0218 (9)	−0.0006 (7)	0.0080 (7)	0.0044 (7)
C3C	0.0299 (10)	0.0311 (10)	0.0198 (9)	0.0011 (8)	0.0099 (8)	0.0050 (7)
C4C	0.0306 (10)	0.0266 (9)	0.0184 (9)	0.0028 (8)	0.0005 (7)	0.0034 (7)
C5C	0.0197 (9)	0.0309 (10)	0.0265 (10)	−0.0001 (7)	0.0016 (7)	0.0036 (8)
C6C	0.0204 (9)	0.0284 (9)	0.0226 (9)	−0.0017 (7)	0.0073 (7)	0.0037 (7)
Cl1C	0.0398 (3)	0.0589 (3)	0.0200 (2)	0.0084 (2)	−0.0012 (2)	0.0080 (2)
P2	0.0134 (2)	0.0174 (2)	0.0147 (2)	−0.00121 (16)	0.00533 (16)	−0.00021 (15)
C1D	0.0142 (8)	0.0171 (8)	0.0202 (8)	0.0000 (6)	0.0082 (6)	0.0003 (6)
C2D	0.0207 (9)	0.0238 (9)	0.0181 (8)	−0.0016 (7)	0.0058 (7)	0.0003 (7)
C3D	0.0218 (9)	0.0221 (9)	0.0237 (9)	−0.0054 (7)	0.0061 (7)	−0.0036 (7)
C4D	0.0172 (8)	0.0166 (8)	0.0298 (9)	0.0009 (7)	0.0120 (7)	0.0022 (7)
C5D	0.0222 (9)	0.0231 (9)	0.0194 (9)	0.0031 (7)	0.0106 (7)	0.0017 (7)
C6D	0.0209 (9)	0.0208 (8)	0.0191 (8)	0.0006 (7)	0.0081 (7)	−0.0029 (7)
Cl1D	0.0312 (2)	0.0191 (2)	0.0334 (2)	−0.00247 (17)	0.01136 (19)	0.00508 (17)
C1E	0.0155 (8)	0.0210 (8)	0.0158 (8)	−0.0002 (6)	0.0064 (6)	0.0038 (6)
C2E	0.0185 (8)	0.0215 (8)	0.0188 (8)	0.0000 (7)	0.0083 (7)	0.0017 (7)
C3E	0.0174 (8)	0.0266 (9)	0.0204 (9)	−0.0032 (7)	0.0047 (7)	0.0016 (7)
C4E	0.0156 (8)	0.0268 (9)	0.0230 (9)	0.0038 (7)	0.0084 (7)	0.0094 (7)
C5E	0.0236 (9)	0.0192 (8)	0.0255 (9)	0.0019 (7)	0.0128 (7)	0.0042 (7)
C6E	0.0206 (9)	0.0210 (8)	0.0203 (8)	−0.0016 (7)	0.0074 (7)	0.0014 (7)
Cl1E	0.0164 (2)	0.0324 (2)	0.0366 (3)	0.00515 (17)	0.01070 (18)	0.00734 (19)
C1F	0.0184 (8)	0.0169 (8)	0.0161 (8)	−0.0005 (6)	0.0059 (6)	−0.0014 (6)
C2F	0.0155 (8)	0.0196 (8)	0.0199 (8)	−0.0013 (6)	0.0062 (7)	−0.0025 (6)
C3F	0.0210 (9)	0.0242 (9)	0.0195 (9)	−0.0040 (7)	0.0094 (7)	−0.0011 (7)
C4F	0.0242 (9)	0.0238 (9)	0.0141 (8)	−0.0017 (7)	0.0031 (7)	−0.0013 (7)
C5F	0.0166 (8)	0.0295 (9)	0.0212 (9)	−0.0012 (7)	0.0036 (7)	−0.0031 (7)
C6F	0.0167 (8)	0.0268 (9)	0.0206 (9)	−0.0024 (7)	0.0077 (7)	−0.0019 (7)
Cl1F	0.0269 (2)	0.0531 (3)	0.0155 (2)	−0.0027 (2)	0.00256 (17)	0.00332 (19)

### Geometric parameters (Å, °)

**Table:**

Fe1—N2	1.6589 (15)	C5C—C6C	1.389 (3)
Fe1—N1	1.6594 (16)	C5C—H5C	0.9500
Fe1—P2	2.2316 (6)	C6C—H6C	0.9500
Fe1—P1	2.2410 (7)	P2—C1F	1.8218 (17)
N1—O1	1.188 (2)	P2—C1E	1.8298 (17)
N2—O2	1.1860 (19)	P2—C1D	1.8362 (17)
P1—C1B	1.8294 (18)	C1D—C2D	1.390 (2)
P1—C1C	1.8322 (18)	C1D—C6D	1.398 (2)
P1—C1A	1.8338 (17)	C2D—C3D	1.390 (2)
C1A—C6A	1.394 (2)	C2D—H2D	0.9500
C1A—C2A	1.401 (2)	C3D—C4D	1.383 (3)
C2A—C3A	1.386 (3)	C3D—H3D	0.9500
C2A—H2A	0.9500	C4D—C5D	1.385 (2)
C3A—C4A	1.383 (3)	C4D—Cl1D	1.7451 (17)
C3A—H3A	0.9500	C5D—C6D	1.382 (2)
C4A—C5A	1.384 (2)	C5D—H5D	0.9500
C4A—Cl1A	1.7390 (18)	C6D—H6D	0.9500
C5A—C6A	1.381 (2)	C1E—C2E	1.392 (2)
C5A—H5A	0.9500	C1E—C6E	1.405 (2)
C6A—H6A	0.9500	C2E—C3E	1.396 (2)
C1B—C6B	1.398 (2)	C2E—H2E	0.9500
C1B—C2B	1.398 (2)	C3E—C4E	1.380 (3)
C2B—C3B	1.387 (2)	C3E—H3E	0.9500
C2B—H2B	0.9500	C4E—C5E	1.392 (3)
C3B—C4B	1.382 (3)	C4E—Cl1E	1.7434 (18)
C3B—H3B	0.9500	C5E—C6E	1.376 (2)
C4B—C5B	1.389 (3)	C5E—H5E	0.9500
C4B—Cl1B	1.7303 (18)	C6E—H6E	0.9500
C5B—C6B	1.384 (2)	C1F—C2F	1.394 (2)
C5B—H5B	0.9500	C1F—C6F	1.400 (2)
C6B—H6B	0.9500	C2F—C3F	1.386 (2)
C1C—C2C	1.393 (2)	C2F—H2F	0.9500
C1C—C6C	1.398 (2)	C3F—C4F	1.383 (2)
C2C—C3C	1.393 (3)	C3F—H3F	0.9500
C2C—H2C	0.9500	C4F—C5F	1.385 (3)
C3C—C4C	1.378 (3)	C4F—Cl1F	1.7394 (18)
C3C—H3C	0.9500	C5F—C6F	1.384 (2)
C4C—C5C	1.387 (3)	C5F—H5F	0.9500
C4C—Cl1C	1.7390 (18)	C6F—H6F	0.9500
			
N2—Fe1—N1	127.78 (7)	C6C—C5C—H5C	120.5
N2—Fe1—P2	106.82 (5)	C5C—C6C—C1C	121.17 (17)
N1—Fe1—P2	104.22 (5)	C5C—C6C—H6C	119.4
N2—Fe1—P1	107.52 (5)	C1C—C6C—H6C	119.4
N1—Fe1—P1	102.22 (5)	C1F—P2—C1E	104.31 (8)
P2—Fe1—P1	106.80 (3)	C1F—P2—C1D	106.01 (8)
O1—N1—Fe1	178.76 (15)	C1E—P2—C1D	98.14 (7)
O2—N2—Fe1	177.67 (14)	C1F—P2—Fe1	113.40 (6)
C1B—P1—C1C	104.80 (8)	C1E—P2—Fe1	121.20 (6)
C1B—P1—C1A	101.59 (8)	C1D—P2—Fe1	111.88 (6)
C1C—P1—C1A	101.95 (8)	C2D—C1D—C6D	118.87 (15)
C1B—P1—Fe1	117.97 (5)	C2D—C1D—P2	124.77 (13)
C1C—P1—Fe1	116.88 (6)	C6D—C1D—P2	116.16 (12)
C1A—P1—Fe1	111.46 (6)	C3D—C2D—C1D	120.61 (16)
C6A—C1A—C2A	118.81 (16)	C3D—C2D—H2D	119.7
C6A—C1A—P1	119.25 (12)	C1D—C2D—H2D	119.7
C2A—C1A—P1	121.86 (13)	C4D—C3D—C2D	119.12 (16)
C3A—C2A—C1A	120.56 (16)	C4D—C3D—H3D	120.4
C3A—C2A—H2A	119.7	C2D—C3D—H3D	120.4
C1A—C2A—H2A	119.7	C3D—C4D—C5D	121.53 (16)
C4A—C3A—C2A	119.19 (16)	C3D—C4D—Cl1D	119.44 (13)
C4A—C3A—H3A	120.4	C5D—C4D—Cl1D	119.03 (14)
C2A—C3A—H3A	120.4	C6D—C5D—C4D	118.73 (16)
C3A—C4A—C5A	121.27 (16)	C6D—C5D—H5D	120.6
C3A—C4A—Cl1A	119.36 (14)	C4D—C5D—H5D	120.6
C5A—C4A—Cl1A	119.36 (14)	C5D—C6D—C1D	121.11 (16)
C6A—C5A—C4A	119.29 (17)	C5D—C6D—H6D	119.4
C6A—C5A—H5A	120.4	C1D—C6D—H6D	119.4
C4A—C5A—H5A	120.4	C2E—C1E—C6E	118.86 (15)
C5A—C6A—C1A	120.86 (16)	C2E—C1E—P2	121.49 (13)
C5A—C6A—H6A	119.6	C6E—C1E—P2	119.62 (12)
C1A—C6A—H6A	119.6	C1E—C2E—C3E	120.62 (16)
C6B—C1B—C2B	118.49 (15)	C1E—C2E—H2E	119.7
C6B—C1B—P1	119.31 (13)	C3E—C2E—H2E	119.7
C2B—C1B—P1	122.14 (13)	C4E—C3E—C2E	118.94 (16)
C3B—C2B—C1B	120.61 (16)	C4E—C3E—H3E	120.5
C3B—C2B—H2B	119.7	C2E—C3E—H3E	120.5
C1B—C2B—H2B	119.7	C3E—C4E—C5E	121.64 (16)
C4B—C3B—C2B	119.51 (16)	C3E—C4E—Cl1E	120.21 (14)
C4B—C3B—H3B	120.2	C5E—C4E—Cl1E	118.15 (14)
C2B—C3B—H3B	120.2	C6E—C5E—C4E	118.92 (16)
C3B—C4B—C5B	121.25 (16)	C6E—C5E—H5E	120.5
C3B—C4B—Cl1B	119.12 (14)	C4E—C5E—H5E	120.5
C5B—C4B—Cl1B	119.63 (14)	C5E—C6E—C1E	121.00 (16)
C6B—C5B—C4B	118.71 (16)	C5E—C6E—H6E	119.5
C6B—C5B—H5B	120.6	C1E—C6E—H6E	119.5
C4B—C5B—H5B	120.6	C2F—C1F—C6F	118.51 (15)
C5B—C6B—C1B	121.40 (16)	C2F—C1F—P2	122.38 (13)
C5B—C6B—H6B	119.3	C6F—C1F—P2	118.41 (13)
C1B—C6B—H6B	119.3	C3F—C2F—C1F	121.13 (16)
C2C—C1C—C6C	118.56 (16)	C3F—C2F—H2F	119.4
C2C—C1C—P1	123.91 (13)	C1F—C2F—H2F	119.4
C6C—C1C—P1	117.52 (13)	C4F—C3F—C2F	118.83 (16)
C3C—C2C—C1C	120.60 (17)	C4F—C3F—H3F	120.6
C3C—C2C—H2C	119.7	C2F—C3F—H3F	120.6
C1C—C2C—H2C	119.7	C3F—C4F—C5F	121.61 (16)
C4C—C3C—C2C	119.60 (17)	C3F—C4F—Cl1F	118.62 (14)
C4C—C3C—H3C	120.2	C5F—C4F—Cl1F	119.76 (14)
C2C—C3C—H3C	120.2	C6F—C5F—C4F	118.90 (16)
C3C—C4C—C5C	121.08 (17)	C6F—C5F—H5F	120.6
C3C—C4C—Cl1C	119.40 (15)	C4F—C5F—H5F	120.6
C5C—C4C—Cl1C	119.51 (15)	C5F—C6F—C1F	121.01 (16)
C4C—C5C—C6C	118.95 (17)	C5F—C6F—H6F	119.5
C4C—C5C—H5C	120.5	C1F—C6F—H6F	119.5
			
N2—Fe1—P1—C1B	50.95 (8)	N2—Fe1—P2—C1F	−161.85 (8)
N1—Fe1—P1—C1B	−172.53 (8)	N1—Fe1—P2—C1F	60.70 (8)
P2—Fe1—P1—C1B	−63.40 (7)	P1—Fe1—P2—C1F	−47.03 (6)
N2—Fe1—P1—C1C	177.35 (8)	N2—Fe1—P2—C1E	−36.62 (8)
N1—Fe1—P1—C1C	−46.13 (8)	N1—Fe1—P2—C1E	−174.07 (8)
P2—Fe1—P1—C1C	63.01 (7)	P1—Fe1—P2—C1E	78.20 (6)
N2—Fe1—P1—C1A	−65.99 (8)	N2—Fe1—P2—C1D	78.31 (8)
N1—Fe1—P1—C1A	70.53 (8)	N1—Fe1—P2—C1D	−59.14 (8)
P2—Fe1—P1—C1A	179.66 (6)	P1—Fe1—P2—C1D	−166.87 (6)
C1B—P1—C1A—C6A	−99.51 (14)	C1F—P2—C1D—C2D	7.28 (17)
C1C—P1—C1A—C6A	152.44 (13)	C1E—P2—C1D—C2D	−100.22 (15)
Fe1—P1—C1A—C6A	27.00 (15)	Fe1—P2—C1D—C2D	131.37 (14)
C1B—P1—C1A—C2A	77.33 (15)	C1F—P2—C1D—C6D	−177.80 (12)
C1C—P1—C1A—C2A	−30.73 (16)	C1E—P2—C1D—C6D	74.70 (14)
Fe1—P1—C1A—C2A	−156.16 (12)	Fe1—P2—C1D—C6D	−53.71 (14)
C6A—C1A—C2A—C3A	0.7 (3)	C6D—C1D—C2D—C3D	−0.4 (3)
P1—C1A—C2A—C3A	−176.11 (13)	P2—C1D—C2D—C3D	174.39 (13)
C1A—C2A—C3A—C4A	0.7 (3)	C1D—C2D—C3D—C4D	−0.6 (3)
C2A—C3A—C4A—C5A	−1.5 (3)	C2D—C3D—C4D—C5D	0.7 (3)
C2A—C3A—C4A—Cl1A	178.34 (13)	C2D—C3D—C4D—Cl1D	−178.72 (13)
C3A—C4A—C5A—C6A	0.9 (3)	C3D—C4D—C5D—C6D	0.3 (3)
Cl1A—C4A—C5A—C6A	−178.98 (13)	Cl1D—C4D—C5D—C6D	179.70 (13)
C4A—C5A—C6A—C1A	0.6 (3)	C4D—C5D—C6D—C1D	−1.3 (3)
C2A—C1A—C6A—C5A	−1.4 (2)	C2D—C1D—C6D—C5D	1.4 (2)
P1—C1A—C6A—C5A	175.54 (13)	P2—C1D—C6D—C5D	−173.83 (13)
C1C—P1—C1B—C6B	−69.90 (15)	C1F—P2—C1E—C2E	126.39 (14)
C1A—P1—C1B—C6B	−175.73 (13)	C1D—P2—C1E—C2E	−124.70 (14)
Fe1—P1—C1B—C6B	62.15 (14)	Fe1—P2—C1E—C2E	−2.92 (16)
C1C—P1—C1B—C2B	113.22 (15)	C1F—P2—C1E—C6E	−55.50 (15)
C1A—P1—C1B—C2B	7.39 (16)	C1D—P2—C1E—C6E	53.40 (14)
Fe1—P1—C1B—C2B	−114.73 (13)	Fe1—P2—C1E—C6E	175.18 (11)
C6B—C1B—C2B—C3B	1.1 (3)	C6E—C1E—C2E—C3E	0.3 (2)
P1—C1B—C2B—C3B	177.96 (14)	P2—C1E—C2E—C3E	178.42 (13)
C1B—C2B—C3B—C4B	0.6 (3)	C1E—C2E—C3E—C4E	0.4 (2)
C2B—C3B—C4B—C5B	−1.7 (3)	C2E—C3E—C4E—C5E	−0.3 (3)
C2B—C3B—C4B—Cl1B	177.93 (14)	C2E—C3E—C4E—Cl1E	179.48 (13)
C3B—C4B—C5B—C6B	1.2 (3)	C3E—C4E—C5E—C6E	−0.6 (3)
Cl1B—C4B—C5B—C6B	−178.48 (13)	Cl1E—C4E—C5E—C6E	179.69 (13)
C4B—C5B—C6B—C1B	0.5 (3)	C4E—C5E—C6E—C1E	1.3 (2)
C2B—C1B—C6B—C5B	−1.6 (2)	C2E—C1E—C6E—C5E	−1.2 (2)
P1—C1B—C6B—C5B	−178.62 (13)	P2—C1E—C6E—C5E	−179.31 (13)
C1B—P1—C1C—C2C	10.70 (17)	C1E—P2—C1F—C2F	−19.21 (16)
C1A—P1—C1C—C2C	116.27 (15)	C1D—P2—C1F—C2F	−122.22 (14)
Fe1—P1—C1C—C2C	−121.97 (14)	Fe1—P2—C1F—C2F	114.65 (13)
C1B—P1—C1C—C6C	−169.53 (13)	C1E—P2—C1F—C6F	170.49 (13)
C1A—P1—C1C—C6C	−63.97 (15)	C1D—P2—C1F—C6F	67.48 (15)
Fe1—P1—C1C—C6C	57.80 (15)	Fe1—P2—C1F—C6F	−55.65 (14)
C6C—C1C—C2C—C3C	0.2 (3)	C6F—C1F—C2F—C3F	1.1 (2)
P1—C1C—C2C—C3C	179.96 (14)	P2—C1F—C2F—C3F	−169.24 (13)
C1C—C2C—C3C—C4C	1.4 (3)	C1F—C2F—C3F—C4F	−1.2 (3)
C2C—C3C—C4C—C5C	−1.4 (3)	C2F—C3F—C4F—C5F	0.1 (3)
C2C—C3C—C4C—Cl1C	177.41 (14)	C2F—C3F—C4F—Cl1F	−179.12 (13)
C3C—C4C—C5C—C6C	−0.2 (3)	C3F—C4F—C5F—C6F	1.1 (3)
Cl1C—C4C—C5C—C6C	−179.01 (15)	Cl1F—C4F—C5F—C6F	−179.67 (14)
C4C—C5C—C6C—C1C	1.8 (3)	C4F—C5F—C6F—C1F	−1.2 (3)
C2C—C1C—C6C—C5C	−1.8 (3)	C2F—C1F—C6F—C5F	0.2 (3)
P1—C1C—C6C—C5C	178.39 (15)	P2—C1F—C6F—C5F	170.88 (14)
